# Cutting-Edge Applications of Cellulose-Based Membranes in Drug and Organic Contaminant Removal: Recent Advances and Innovations

**DOI:** 10.3390/polym16202938

**Published:** 2024-10-20

**Authors:** Bandana Padhan, Wanki Ryoo, Madhumita Patel, Jatis Kumar Dash, Rajkumar Patel

**Affiliations:** 1 Department of Biotechnology, School of Life Science and Biotechnology, Adamas University, Kolkata 700126, India; miki.bandana@gmail.com; 2 Bio-Convergence, Integrated Science and Engineering Division, Underwood International College, Yonsei University, 85 Songdogwahak-ro, Yeonsu-gu, Incheon 21983, Republic of Korea; 3 Department of Chemistry and Nanoscience, Ewha Womans University, 52 Ewhayeodae-gil, Seodaemun-gu, Seoul 03760, Republic of Korea; madhurk29@gmail.com; 4 Department of Physics, SRM University-AP, Amaravati 522502, India; 5 Energy and Environmental Science and Engineering (EESE), Integrated Science and Engineering Division (ISED), Underwood International College, Yonsei University, Incheon 21983, Republic of Korea

**Keywords:** cellulose-based membrane bioreactor, industrial effluents, antibiotics removal, water pollution, wastewater treatment, human health, sustainability

## Abstract

The increasing environmental challenges caused by pharmaceutical waste, especially antibiotics and contaminants, necessitate sustainable solutions. Cellulose-based membranes are considered advanced tools and show great potential as effective materials for the removal of drugs and organic contaminants. This review introduces an environmentally friendly composite membrane for the elimination of antibiotics and dye contaminants from water and food, without the use of toxic additives. The potential of cellulose-based membranes in reducing the impact on water quality and promoting environmental sustainability is emphasized. Additionally, the benefits of using biobased cellulose membranes in membrane biological reactors for the removal of antibiotics from pharmaceutical waste and milk are explored, presenting an innovative approach to achieving a circular economy. This review provides recent and comprehensive insights into membrane bioreactor technology, making it a valuable resource for researchers seeking efficient methods to break down antibiotics in industrial wastewater, particularly in the pharmaceutical and dairy industries.

## 1. Introduction

In recent years, there has been a growing global concern about the threat posed by emerging contaminants to both aquatic ecosystems and human health. Notably, pharmaceutical residues (PhaRs) have attracted considerable attention due to their widespread presence in various environmental settings, including surface water, groundwater, and wastewater treatment plant effluents [1,2]. The diverse range of PhaRs, encompassing steroids, antibiotics, antidepressants, antacids, and analgesics, presents a significant environmental challenge that is exacerbated by insufficient monitoring, regulatory frameworks, validated analytical methods, and comprehensive information regarding their prevalence. Despite their individual non-persistence, pharmaceutical residues accumulate in the environment, creating a pseudo-persistent presence that poses substantial risks to aquatic life and human health [3]. The ecological risks associated with PhaRs extend to disrupting the food chain, with various species, such as bacteria, algae, and invertebrates, experiencing adverse effects even at low concentrations. Nevertheless, the limited research in this area underscores the pressing need for innovative technologies tailored to effectively remediating these organic micropollutants [4].

Similarly, organic dyes represent another category of emerging contaminants that contribute significantly to water pollution, primarily through effluents from industries such as textiles and paper [5]. These dyes have been found to have severe toxic effects on aquatic life, affecting water quality and the overall health of aquatic environments. These dyes impair water transparency, which subsequently reduces solar radiation penetration and negatively affects photosynthesis in aquatic flora [6]. Additionally, the accumulation of these dyes and associated contaminants in the food chain can pose significant risks to agricultural productivity, affecting crop quality and soil health [7]. Pesticides and herbicides are synthetic organic compounds that are widely used in agriculture to control pests and weeds. Commonly, glyphosate and atrazine are used as herbicides; they have been detected in water sources and impact the reproductive and developmental processes in aquatic life. The lack of extensive research in this field underscores the necessity of developing advanced methodologies and frameworks that can adequately address the presence of diverse organic micropollutants, including PhaRs and organic dyes, in our ecosystems [4].

One effective approach to tackling contaminants of emerging concern in both wastewater and drinking water is membrane technology, which has become a focal point in recent research [3,8]. In particular, cellulose-based membranes have emerged as promising materials for water remediation due to their abundance, biocompatibility, and large surface area. These membranes can be used for adsorption, catalysis, and anti-fouling applications in water treatment. These membranes exhibit variable pore architectures, which allows for the effective separation of different contaminants. Various fabrication methods, such as phase inversion and the use of cellulose particles, enable the adjustment of the pore size and porosity. One study demonstrated a composite cellulose-based membrane with an average pore size of 2.2 nm, achieving a flux of 12 L m^−2^ h^−1^ and a sodium chloride rejection rate of 36%. This tunable porosity allows these membranes to effectively target a range of pollutants, including organic molecules and heavy metals, thus enhancing their efficacy in water treatment [9]. It has been noted that cellulose membranes can exhibit excellent selective separation capabilities for various ions and organic compounds, which is critical for applications such as ultrafiltration and reverse osmosis. The mechanical strength of cellulose-based membranes can be enhanced through the incorporation of cellulose nanocrystals (CNCs) or cellulose nanofibrils (CNFs). These nanomaterials contribute to significant increases in tensile strength, making membranes more robust. For instance, a 0.8% CNF-doped polysulfone membrane showed an increase in tensile strength from 158 MPa to 391 MPa compared to a neat polysulfone membrane. The high mechanical stiffness of CNCs and CNFs, alongside cellulose’s own impressive tensile properties, ensure that these membranes withstand the stresses of filtration processes while maintaining their function [10].

Cellulose-based membranes have gained attention as promising materials for water remediation due to their abundance, biocompatibility, and large surface area. These membranes can be utilized for various applications in water treatment, such as adsorption, catalysis, and anti-fouling. For example, composite cellulose acetate/layered double hydroxide (LDH) membranes have been successfully employed for the removal of diclofenac sodium and tetracycline from water [11]. With their porous structures, these membranes enable the efficient filtration of organic contaminants including pharmaceutical residues, pesticides, and dyes. Moreover, cellulose-based materials can be chemically and physically modified to enhance their mechanical properties and improve their water treatment capabilities. For instance, carboxyl groups, sulfonate groups, and phosphonate groups can be grafted onto nanocellulose to selectively uptake contaminants in water remediation [12]. These membranes find extensive use in water treatment processes, offering versatile solutions for the removal of organic contaminants [13,14,15,16]. The current research is focused on optimizing cellulose membranes’ properties for specific applications, with the integration of nanotechnology showing the potential to further enhance their performance, particularly in addressing challenges posed by pharmaceutical residues and other organic contaminants [17,18]. In recent years, researchers have explored various innovative techniques and materials for the efficient removal of tetracycline residues from water and food products [19]. These include advanced membrane technologies such as the use of Cu_2_(OH)_2_CO_3_/g-C_3_N_4_ in cellulose acetate (Cu/CN@CA) composite membranes, mixed-mode strong cation-exchanging fabric phase sorptive extraction (FPSE) techniques [2], amino-functionalized Fe-based metal–organic frameworks [3], and photocatalytic membranes [8,20]. These approaches demonstrate promising capabilities in removing tetracycline residues from both aqueous solutions and food matrices, thereby contributing to environmental and food safety.

The quality of water and food products is incredibly important in maintaining environmental sustainability and public health [21,22]. Contamination from substances like antibiotics and dyes presents significant challenges to water and food safety, requiring new approaches for their removal [20,23,24]. Previous reports have discussed customizing nanocellulose’s surface chemistry for pollutant removal and membrane fabrication, as well as the potential of nanocellulose in improving water filtration [25,26,27]. Another review focused on cellulose derivatives, highlighting their mechanisms of adsorption and effectiveness in removing pollutants such as heavy metals, dyes, and pathogens from wastewater [18,28,29]. This review thoroughly explores various methodologies and materials aimed at addressing water and food contamination, with a particular focus on removing tetracycline, other antibiotics, dye contaminants, and other pollutants. Furthermore, the review discusses emerging techniques for the removal of contaminants of various kinds, including polyacrylonitrile nanofiber membranes for organic micropollutant removal, cellulose acetate/zeolite fiber adsorbents for erythromycin removal, and recycled polyvinyl chloride microplastics for antibiotic adsorption. This review presents groundbreaking insights that distinguish it from other literature in the field by introducing novel extraction techniques and emphasizing critical issues related to environmental contaminants. Notably, it discusses the development of mixed-mode solid-phase extraction (FPSE) and amino-functionalized metal–organic frameworks (MOFs), which offer unprecedented sensitivity and efficiency in detecting and removing antibiotic residues in food products and wastewater. Unlike many existing reviews that broadly address environmental pollution, this work specifically highlights dye contaminants in water, focusing on their origins in industrial processes such as textile, paper, and food processing. Additionally, it showcases innovative advancements like water-stable membranes produced from carboxymethyl cellulose (CMC) and cellulose nanofibrils (CNFs), cross-linked with citric acid, emphasizing their potential in enhancing contaminant removal. Furthermore, the exploration of nanocomposite membranes derived from cellulose nanocrystals (CNCs) reflects significant technological progress, offering enhanced performance for contaminant extraction. By integrating these diverse approaches, this review provides a comprehensive perspective on environmental sustainability and public health protection, thereby serving as a crucial resource for researchers in the field. Its focus on innovative methodologies and specific contaminant challenges sets it apart from traditional reviews, paving the way for future research and practical applications.

## 2. Conventional Membranes vs. Cellulosic Membranes: Limitations and Challenges

Conventional membrane techniques such as nanofiltration, reverse osmosis, and forward osmosis are effective for specific water treatment applications. Nanofiltration membranes are specifically designed to remove small organic molecules and multivalent ions from water, making them widely used in water softening and wastewater treatment. However, NF membranes face limitations such as susceptibility to fouling, particularly from organic substances, which reduces their efficiency and increases the maintenance costs. The high pressure required for the NF process also elevates the energy consumption, making it less sustainable for large-scale applications [30]. Furthermore, NF is less effective in removing monovalent ions, limiting its use in desalination. In comparison, cellulosic membranes offer better biocompatibility and are less prone to fouling due to their hydrophilic nature, although they generally have lower selectivity and mechanical strength compared to NF membranes [31].

Reverse osmosis is one of the most effective membrane filtration methods, capable of removing dissolved salts, organic compounds, and even small contaminants such as viruses. Despite its high efficiency, RO systems have significant drawbacks, including high energy consumption due to the high pressure needed for effective filtration. Additionally, RO membranes are prone to fouling and scaling caused by organic compounds and dissolved salts, reducing their performance. Another major challenge with RO is the disposal of concentrated brine byproducts, which is costly and difficult to manage, especially in inland areas. In contrast, cellulosic membranes are more environmentally friendly and less energy-intensive, but they are not as effective as RO in desalination or removing small contaminants [32].

Forward osmosis is a membrane-based technology that uses osmotic pressure differences to drive water across a semi-permeable membrane, making it a more energy-efficient alternative to reverse osmosis. However, FO faces several challenges, including the recovery of the draw solution, which can be complex and energy-intensive. FO membranes, while less prone to fouling than RO and NF membranes, still suffer from fouling issues. Moreover, FO generally has lower water flux compared to RO, making it less efficient for large-scale desalination projects. Cellulosic membranes, while offering lower performance in handling high-salinity solutions, are biodegradable and environmentally friendly. However, FO membranes’ complex draw solution recovery requirements are not an issue for cellulosic membranes, which makes them more suitable for sustainable applications [33].

Cellulosic membranes, derived from renewable plant-based materials, offer a biodegradable and environmentally sustainable alternative to synthetic membranes such as those produced from polyamide and used in nanofiltration (NF), reverse osmosis (RO), and forward osmosis (FO). Their eco-friendly nature significantly reduces the environmental impact associated with membrane disposal, making them a greener option for water treatment. Fouling, caused by the accumulation of organic and inorganic substances, leads to reduced efficiency and higher maintenance costs in NF, RO, and FO. Cellulosic membranes, with their lower fouling potential, boast longer operational lifetimes and require less frequent cleaning, ultimately lowering the maintenance expenses. Additionally, cellulosic membranes are more energy-efficient, particularly in applications where lower-pressure conditions are sufficient, unlike RO systems that require high pressure and significant energy to function. As a sustainable solution aligned with environmental goals, these membranes offer a clear advantage over petroleum-based synthetic membranes, which contribute to environmental degradation and a reliance on non-renewable resources [34].

## 3. Removal of Drugs from the Water

### 3.1. Removal of Tetracycline

Tetracycline is an antibiotic used to treat a variety of bacterial infections in humans and animals. It is classified under the tetracycline class of antibiotics and works by inhibiting bacterial protein synthesis, effectively controlling the growth of bacteria [35]. Tetracycline, an antibiotic used in humans and animals, contaminates water and food products through agricultural runoff, wastewater discharges, and improper pharmaceutical disposal. Agricultural practices, particularly the use of tetracyclines as growth promoters in livestock, lead to residue runoff, while ineffective wastewater treatment from farms, hospitals, and manufacturing allows tetracycline to enter water bodies. The improper disposal of unused antibiotics also contributes to contamination [36]. This contamination poses risks to aquatic ecosystems, disrupting microbial communities, reducing biodiversity, and fostering antibiotic resistance. For humans, the consumption of contaminated water or food can cause allergic reactions, antibiotic-resistant infections, and adverse effects on fetal development [37].

The proper treatment of tetracycline residues in water and food products is crucial in ensuring environmental and food safety. Various innovative methods and materials have been investigated to address tetracycline contamination [38]. Recent research has explored the removal of tetracycline from water using cellulose-based membranes. Studies have also revealed promising developments in the synthesis and application of advanced membranes for efficient and sustainable water treatment. Advanced approaches such as basic copper carbonate/graphitic carbon nitride (Cu_2_(OH)_2_CO_3_/g-C_3_N_4_) heterojunctions on cellulose acetate composite membranes (Cu/CN@CA) and polypyrrole (PPy)-covered bacterial cellulose (BC)/graphitic carbon nitride (g-C₃N₄) flexible nanofiber membranes (PPy@BC/g-C_3_N_4_) represent innovative solutions for the removal of typical dyes, antibiotics, and trace organic pollutants [2,20]. A mixed-mode strong cation-exchanging fabric phase sorptive extraction (FPSE) technique has been reported for the determination of tetracycline residues in milk through protein precipitation. This method utilizes a stable sorbent with a unique extraction membrane composed of sol–gel C18/propyl sulfonic acid on a cotton cellulose fabric substrate and has been comprehensively validated. Parameters such as the linearity, selectivity, stability, limit of detection/limit of quantification (LOD/LOQ), decision limit, detection capability, trueness, precision, and ruggedness were systematically assessed. The recovery of tetracycline residues displayed a range of 88.9% to 122.4%, with the LOD and LOQ determined at 15 μg/kg and 50 μg/kg, respectively [1]. Additionally, iron(III)-loaded cellulose nanofibers (Fe(III)@CNFs) derived from bamboo have been developed for the removal of tetracycline, chlortetracycline, and oxytetracycline from aqueous solutions [39]. The Fe(III)@CNFs adsorbents showed high adsorption capacities for TCs, with surface complexation between the Fe(III) and TCs being the dominant adsorption mechanism [39]. In another study, a novel amino-functionalized Fe-based metal–organic framework, NH_2_-MIL-88B, was highly effective in extracting tetracycline from milk. The introduced amino groups formed hydrogen-bonding interactions with the hydroxyl groups of tetracycline, enhancing the peroxidase-like activity and facilitating electron transfer, promoting the Fenton reaction. The NH_2_-MIL-88B-based sensor exhibited excellent selectivity and a low limit of detection, showcasing its potential for rapid and sensitive TC detection in milk samples. The combination with cellulose acetate to create nanozyme hybrid membranes holds promise for the development of point-of-care testing devices, particularly in the context of food safety and analytical chemistry applications [3].

**Figure 1:**
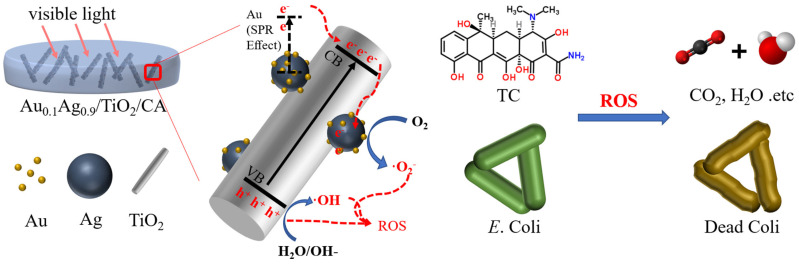
Mechanism of tetracycline degradation and antibacterial action in Au0.1Ag0.9/TiO_2_/CA membrane [40].

A photocatalytic membrane (Au0.1Ag0.9/TiO_2_/CA) has been created to break down antibiotics, specifically tetracycline, and remove harmful bacteria from water. This membrane, which combines bimetallic nanoparticles with TiO_2_ nanorods in cellulose acetate, has shown great effectiveness in breaking down tetracycline when exposed to visible light (Figure 1). The photocatalytic process used by the Au0.1Ag0.9/TiO_2_/CA membrane provides a solution for the removal of both antibiotics and bacteria from water simultaneously. This development has promising applications in water treatment and offers a way to reduce antibiotic pollution in aquatic environments [40].

The use of cellulose nanocrystalline (CNC) materials, functionalized cellulose acetate membranes, and innovative nanocomposite membranes reflects dedication to environmentally friendly and cost-effective methods of purifying contaminated water [13,41,42]. Sulfated cellulose nanocrystalline composite membranes were created to remove tetracycline hydrochloride from water. The CNC materials, which were produced from microcrystalline cellulose, displayed effective removal capabilities when incorporated into composite membranes. The combined effect of steric hindrance and electrostatic interaction within the composite membranes led to improved removal efficiency, providing an environmentally friendly solution to address antibiotic pollution and water purification [13].

In an effort to efficiently remove tetracycline from aqueous solutions, cellulose acetate membranes were modified using aminopropyl triethoxysilane. This process involved reacting the remaining free amino groups with cyanuric chloride in a heated feed solution containing tetracycline. This reactive retention method significantly increased the adsorption capacity from 16% to an impressive 88%. Additionally, the modified membrane showed a substantial improvement in permeability, with the flux rate increasing from 72 L/m^2^·h to 517.66 L/m^2^·h compared to the pure cellulose acetate membrane [41]. To address the issue of residual pharmaceutical contaminants in wastewater, a novel approach involved creating cellulose acetate/Mg-Al layered double hydroxide (Mg-Al LDH) nanocomposite membranes for the efficient removal of pharmaceutical substances. These membranes were assessed for their hydrodynamic properties and adsorption capacity using both pure water and aqueous solutions containing common drugs, diclofenac sodium (DS), and tetracycline (TC). The nanocomposite membranes exhibited improved permeability compared to plain cellulose acetate. Particularly notable was the membrane containing 4 wt.% Mg-Al LDH loading, demonstrating the highest water flux at 529 L/m^2^·h, a significant increase compared to 36 L/m^2^·h in the pure membrane. Moreover, this membrane showcased a tenfold increase in the adsorption capacity for DS, attributed to electrostatic interactions, and a considerable enhancement in the TC adsorption capacity due to hydrogen-bonding interactions with the nanofiller. These approaches offer valuable insights and potential solutions to address the challenge of pharmaceutical contaminants in wastewater [42].

A highly efficient and sustainable Cu/CN@CA composite membrane was developed for the removal of dyes and antibiotics simultaneously. The membrane, which incorporates a basic copper carbonate (Cu₂(OH)₂CO₃) and graphitic carbon nitride (g-C₃N₄) (Cu_2_(OH)_2_CO_3_/g-C_3_N_4_) heterojunction (Cu/CN) onto a cellulose acetate (CA) substrate, exhibited exceptional adsorption capacities. In particular, the 0.2Cu/CN@CA membrane, optimized with Cu/CN doping, showed remarkable removal rates for Congo red (CR) and tetracycline (TC) at 250.8 mg/g and 48.43 mg/g, respectively. The cellulose acetate component played a key role in facilitating charge transfer interactions between g-C_3_N_4_ and Cu_2_(OH)_2_CO_3_, establishing a type II heterojunction transfer pathway. This mechanism induced a strong oxidizing capability in the membrane, leading to the generation of active species for the photocatalytic degradation of adsorbed contaminants under solar light exposure [2].

Researchers have also explored the potential of photocatalytic technology for the effective removal of trace organic pollutants, such as tetracycline hydrochloride, from water [20]. One challenge in photocatalysis is the separation of the photocatalyst from the water after use, especially when using powder photocatalysts. To address this issue, the researchers employed a different approach by uniformly assembling graphitic carbon nitride (g-C_3_N_4_) on bacterial cellulose (BC), forming a 3D nanofiber network, and enhancing its catalytic activity through the incorporation of polypyrrole (PPy). The catalytic activity was further enhanced by incorporating polypyrrole (PPy), resulting in the development of the flexible membrane known as PPy@(BC/g-C_3_N_4_). This membrane exhibited remarkable catalytic efficiency of 64.28% for TC-H degradation over 2 h when exposed to low-power xenon lamp irradiation (λ > 420 nm) in a specially designed reactor. Notably, the PPy@(BC/g-C_3_N_4_) membrane demonstrated excellent stability, retaining more than 80% of its initial catalytic activity even after ten cycles of repeated use. This approach introduces a general strategy for the development of flexible membrane materials with high-efficiency catalytic properties [20].

Furthermore, in a study, a zeolitic imidazole framework-8 (ZIF-8) was incorporated into a cellulose membrane (ZIF-8/CM) and installed in a dismountable filter for use as a sorbent in a lab-made microextraction in packed syringe (MEPS) system. The MEPS procedure was simplified to a filtration process since the macroporous cellulose membrane served as the substrate for ZIF-8 powder. The combined ZIF-8/CM-MEPS system, coupled with high-performance liquid chromatography and a tandem ultraviolet detector (HPLC-UV), was employed for the analysis of tetracyclines in water samples [4].

**Figure 2:**
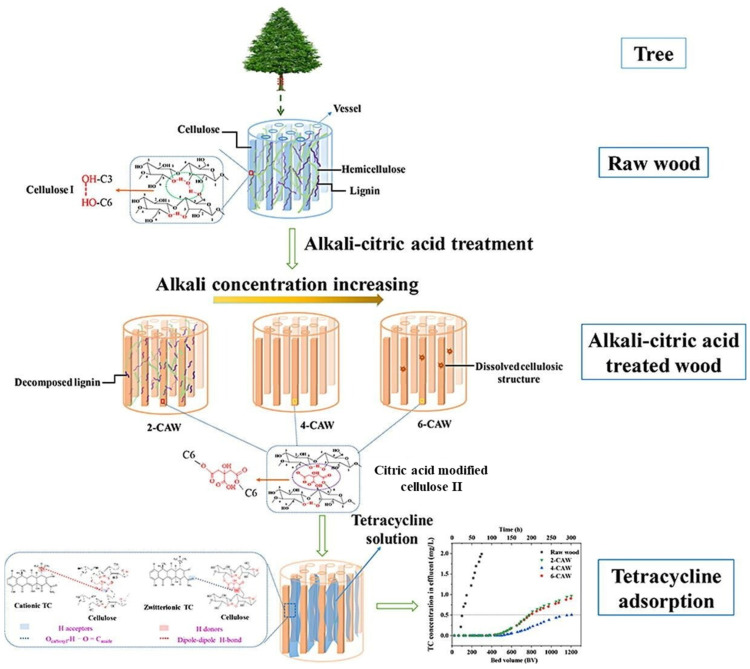
Schematic illustration of x-CAW synthesis and tetracycline adsorption on raw wood and x-CAW [43].

Wood membranes (WMs) are promising materials for environmental remediation, especially in addressing antibiotic contaminants like tetracycline (TC) in water. In a study, WMs were modified with sodium hydroxide (NaOH) and esterified with citric acid (CA) to enhance the TC adsorption capacity. The x-CAW membranes showed breakthrough curves with 8–12 times greater effective treatable volumes and higher adsorption capacities compared to raw wood (Figure 2). Notably, significant TC adsorption occurred at pH 5 due to the hydrogen bonds formed between the O_carboxyl_–H group of x-CAW and the O=C_amide_ group of zwitterionic TC [43].

In another study, wood membranes were modified with citric acid (CA) to create cost-effective and efficient adsorption membranes for the removal of TC. The modification of pinewood (PW) and basswood (BW) transformed the structure of the wood membranes, resulting in enhanced surface properties. The study found that the 6 wt% alkali-CA modified BW showed a significantly greater effective filtration volume compared to the 4 wt% alkali-CA modified PW. The study also highlighted the influence of the pH on TC adsorption, with zwitterionic TC showing favorable adsorption at around pH 5. However, as the pH increased, the TC adsorption efficiency decreased due to electrostatic repulsion [44].

In a parallel development, a multifaceted approach, encompassing modification techniques and innovative materials like non-cytotoxic fluorescent wood (NCFW), has opened up new avenues for sustainable and effective solutions in water treatment. NCFW demonstrated an impressive TC adsorption capacity, efficiently removing up to 99.17 mg/g of TC from water. The unique structure of NCFW improved TC adsorption, and integrated fluorescent probes enhanced its effectiveness as a sensor. With an absolute quantum yield of 3.9%, the NCFW exhibited notable selectivity and sensitivity for TC detection, making it a promising multifunctional solution for water samples. Importantly, the NCFW proved to be non-cytotoxic, with cell viability of over 90%, ensuring its safety for diverse applications [45].

### 3.2. Removal of Ciprofloxin

The use of various types of cellulose acetate nanofiber membrane systems for the removal of ciprofloxacin (CIP) from water represents a significant advancement in wastewater treatment technology [46,47]. These membranes show strong adsorption characteristics, especially at pH levels where CIP is in its zwitterionic form. Additionally, their endothermic nature and increased effectiveness at higher temperatures make them applicable in various environmental conditions [48]. The use of different types of cellulose acetate nanofiber membrane systems for CIP removal indicates a promising approach to improving water quality and addressing environmental challenges associated with antibiotic contamination [14,49]. Several studies highlight the importance of employing various nanomaterial-based approaches for the efficient and sustainable removal of CIP from water, addressing critical environmental challenges while ensuring effectiveness in water treatment processes [23,50].

The removal of ciprofloxacin from water has been addressed through various innovative approaches, each offering distinct advantages in terms of efficiency and sustainability. Initially, montmorillonite-impregnated cellulose acetate nanofiber membranes (MMT-CA-NFM) were used for CIP adsorption. Batch experiments revealed optimal conditions for adsorption, with a 76% removal rate achieved at a CIP concentration of 10 mg/L, an MMT-CA-NFM dose of 4 g/L, and a pH of 6.0. The zwitterionic form of CIP was found to enhance the adsorption efficacy, particularly within a pH range of 6–7.5, and higher temperatures facilitated the endothermic adsorption process. Regeneration studies showed that CIP could be desorbed using a 10 mM NaOH solution, with the nanofibers retaining their adsorption capacity after multiple uses, indicating their potential for repeated application in water treatment [14]. Another approach involved the use of magnetic nanosorbents, where sulfated polysaccharides combined with an epoxide-ring-containing alkoxysilane agent were employed. This method showed exothermic and entropically favorable adsorption, with κ-carrageenan-based sorbents showing spontaneous adsorption at room temperature. Reusability tests confirmed the maintenance of the adsorptive performance, suggesting their efficiency in wastewater treatment [49].

Studies have shown that magnetic nanosorbents produced with sulfated polysaccharides and modified graphene-oxide-functionalized cellulose nanofibers are effective in adsorbing CIP. These nanoadsorbents can efficiently adsorb ciprofloxacin and ofloxacin, with maximum removal capacities of 45.04 mg/g and 85.30 mg/g, respectively. They can be regenerated and reused, making them cost-effective and environmentally friendly for the removal of pharmaceutical pollutants [50]. In addition, empirical models using magnetized functionalized multi-walled carbon nanotubes (FMWCNTs-Fe_3_O_4_) can predict the CIP removal efficiency. The Langmuir model suggests a high maximum adsorption capacity of 107.66 mg/g, with the adsorption kinetics following the pseudo-second-order model. The FMWCNTs-Fe_3_O_4_ also demonstrate good reusability, with only a 12% decrease in efficiency after five cycles, showing their effectiveness in treating water contaminated with CIP [23].

### 3.3. Removal of Other Antibiotics

A number of studies have investigated innovative methods to address the urgent problem of antibiotic pollution in water. These methods offer promising solutions for the efficient removal of antibiotics and other contaminants, thereby contributing to improved water quality and environmental sustainability [51]. These studies demonstrate the diverse range of approaches and materials being explored for the efficient removal of antibiotics and other contaminants from water, offering promising solutions to address water quality challenges and environmental sustainability [14,19,24,52].

In one study, colemanite was demonstrated as a novel adsorbent for fluoroquinolone antibiotics from wastewater. The adsorption process was found to be spontaneous and endothermic, with the effectiveness increasing with changes in the pH and temperature. Notably, colemanite demonstrated efficacy and reusability over multiple cycles, suggesting its potential as a repeatable adsorbent for fluoroquinolones and similar pollutants [24]. Additionally, a viscose/bacterial cellulose (BC) composite membrane embedded with graphene oxide (GO) was utilized to treat sulfonamide antibiotic wastewater. The composite membrane, coupled with an enzyme carrier with high efficiency, demonstrated high removal rates for sulfonamides under specific operational conditions. This scalable strategy presents a transformative approach to treating and recycling antibiotic wastewater [19]. Moreover, semiconductor photocatalysis and membrane separation were integrated into water treatment technology using ultralong nanowires (UNWs) of the Ca–alendronate (Ca-ALN) complex. This multifunctional photocatalytic filter paper, capable of photocatalysis, adsorption, and filtration, exhibited high pure water flux rates, indicating its potential for efficient water purification and biomedical applications [52].

In the field of wastewater treatment, membranes derived from two-dimensional (2D) materials such as laminated graphene oxide (GO) and cellulose nanocrystal hybrids have shown promise. By combining GO and CNCs, these membranes exhibit improved surface hydrophilicity, a crumpled structure, and slightly increased interlayer spacing, which significantly enhances the water permeability (two to four times higher than pure GO membranes). The optimal GO/CNC membrane demonstrated high antibiotic rejection rates of 74.8% for sulfamethoxazole (SMX), 90.9% for levofloxacin (Levo), and 97.2% for norfloxacin (Nor), while allowing the passage of essential nutrients such as NO_3_^−^ and H_2_PO_4_^−^. The removal mechanisms included electrostatic repulsion for SMX and adsorption for Levo and Nor [53]. In addition, heterostructures of reduced graphene oxide–tungsten trioxide (rGO-WO_3_) have proven to be efficient adsorbents for the removal of contaminants like levofloxacin from water. The integration of WO_3_ nanorods onto rGO nanosheets significantly increased their adsorption capacities, demonstrating their potential for water treatment applications [54].

**Figure 3:**
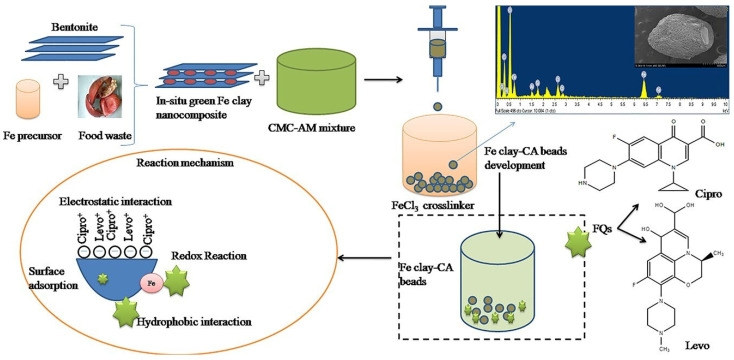
Mechanism of adsorptive removal of fluoroquinolone antibiotics using green-synthesized and highly efficient Fe clay cellulose–acrylamide beads [55].

A hybrid photocatalytic membrane, which integrated graphitic carbon nitride (g-C_3_N_4_) nanosheets with graphene oxide (GO) interlayers, was employed for effective wastewater treatment. The membrane’s performance was tested in reactor cells using organic dyes and antibiotics as test pollutants, and it was assessed based on its permeance, rejection rate during filtration, overall removal efficiency under light irradiation, and degradation rate. The membrane demonstrated improved permeance under visible light, high stability over repeated use, and consistently high removal rates for dyes and various antibiotics [8]. Additionally, a separate study reported the use of clay polymeric nanocomposite hydrogel beads in adsorbing fluoroquinolone antibiotics from water. These beads, produced from carboxymethyl cellulose, acrylamide, and Fe clay through an ionotropic gelation process (refer to Figure 3), removed approximately 92% of ciprofloxacin and 93% of levofloxacin, with maximum adsorption capacities of 57.84 mg/g for ciprofloxacin and 38.01 mg/g for levofloxacin. Furthermore, this treatment significantly reduced the residual toxicity against freshwater algae *Chlorella* spp., underscoring the potential environmental benefits of this method in reducing antibiotic pollution [55].

Furthermore, a novel two-step immobilization method was developed to produce cross-linked enzyme aggregates (CLEA) within membrane pores. Laccase was adsorbed onto a biodegradable cellulose acetate membrane and cross-linked to enhance the enzyme loading and efficiency. The resultant membrane had 76% immobilization efficiency at 29 °C, with surface activity of 1174 U/m^2^, despite some denaturation and membrane fouling. The immobilized laccase achieved 58% removal efficiency for diclofenac from wastewater [56]. Similarly, the remediation of penicillin antibiotics from wastewater can be achieved through various advanced treatment technologies and methods. Key approaches include biological processes, adsorption techniques, and physical separation processes [57,58]. In a study, aluminum-based metal–organic framework (Al-MOF) polymer monoliths were prepared via microwave-assisted polymerization, using MIL-53(Al) combined with ethylene dimethacrylate (EDMA) and butyl methacrylate (BMA). These Al-MOF polymer monoliths were used as sorbents in solid-phase microextraction (SPME) to extract penicillins (penicillin G, V, oxacillin, cloxacillin, dicloxacillin, and nafcillin). The MIL-53(Al) polymer achieved the highest extraction recoveries (90.5–95.7%), with good reproducibility (<4.2% RSDs). Its application in spiked river water and milk samples yielded recoveries of 80.8–100.7% with <7.1% RSDs, demonstrating its effectiveness in penicillin extraction from environmental and food samples [59]. Various methodologies exist for the remediation of another group of broad-spectrum antibiotics of the β-lactam family, namely cephalosporin antibiotics from wastewater, utilizing biochemical, chemical, and physical treatment processes [60,61]. The removal of the cephalosporin antibiotic cefadroxil was achieved using polyethylenimine (PEI) cross-linked nanofiltration (NF) membranes, as demonstrated by Zhao et al. [62]. These membranes were developed by modifying P84 co-polyimide precursor membranes through cross-linking with PEI of varying molecular weights. This study introduced the concept of the separation behavior of positively charged NF membranes for the removal of antibiotics under different pH conditions. However, no comprehensive research has been conducted specifically on the use of cellulose membrane technology for the removal of penicillin and cephalosporin from wastewater.

Macrolides are a class of antibiotics derived from *Saccharopolyspora*, a genus of soil-borne bacteria. These antibiotics are characterized by their macrocyclic lactone ring structure, which is crucial for their antibacterial activity. Common examples of macrolides include picromycin, clarithromycin, azithromycin, and erythromycin. They are widely used to treat various bacterial infections due to their ability to inhibit protein synthesis in bacteria, making them effective against a broad range of pathogens [63]. Macrolide pollution in wastewater primarily originates from various sectors, notably human and veterinary medicine, pharmaceutical manufacturing, agricultural runoff, and hospital waste. Conventional activated sludge (CAS) treatment has been shown to reduce macrolide residues in wastewater but is not entirely effective in removing them [64]. Membrane bioreactor (MBR) systems, however, have demonstrated higher efficiency in removing macrolides and other antibiotics, with studies showing an average of around 20% greater removal compared to CAS treatment [65]. Despite the improvements seen with MBR systems, the removal of macrolides from wastewater could be further enhanced through advanced membrane technologies, particularly cellulose-based membranes. However, there is a notable gap in the literature regarding the use of cellulose membranes for macrolide remediation, despite their many advantages. This highlights the need for further research and the implementation of cellulose-based membranes in wastewater treatment to effectively tackle macrolide pollution.

## 4. Removal of Dye Contaminants from Water

Dye contaminants in water primarily originate from various industrial processes, particularly in textile industries, paper mills, and food processing plants. Each of these sectors employs dyes for different purposes, leading to significant environmental impacts due to the improper discharge of dye-laden effluents. Textile dyeing is responsible for approximately 20% of all industrial water pollution [66]. Approximately 10,000 different dyes are utilized in this sector, with around 60–70% being azo dyes, which are known carcinogens. During the dyeing process, it is estimated that 10–50% of the dye used can end up in wastewater, significantly contributing to environmental contamination. The textile industry consumes large amounts of water, often up to 200 tons per ton of dyed fabric. Consequently, millions of gallons of effluent, rich in colors and organic chemicals, are discharged as hazardous waste [66,67]. Paper mills are another major source of dye contaminants in water. Effluents from these facilities often contain unutilized dyes such as azo, phthalocyanine, and anthraquinone dyes, which are typically harmful. The wastewater discharged from paper mills is highly toxic and has been reported to contain over 250 different chemicals, which contribute to aquatic pollution and pose significant risks to water quality [7]. The food industry also utilizes synthetic dyes for various applications, contributing to dye contamination. These dyes can leach into wastewater during processing and can be harmful not only to the environment but also to human health [67].

Dye contamination in water causes discoloration, reducing sunlight penetration and hindering photosynthesis, which disrupts aquatic ecosystems by inhibiting essential microorganisms at the base of the food chain. Toxic dyes interfere with aquatic life, affecting growth and reproduction, while some ionic dyes cause skin and respiratory issues in fish and invertebrates [68]. Carcinogenic dyes further contribute to ecological imbalances, and bioaccumulation in fish poses health risks to humans who consume contaminated species. For humans, dye-contaminated water can cause allergies, skin diseases, asthma, and nervous system disorders. Non-biodegradable textile dyes can lead to chronic conditions, including cancer, even at low concentrations [69].

The removal of dye contaminants from wastewater is vital for water purification processes, and several innovative methods have been developed to effectively address this challenge. Advanced membrane technologies and surface modifications offer promising solutions for the efficient removal of dye contaminants from wastewater [70]. Studies have demonstrated the effectiveness of cellulose-based membranes in removing dyes from water, with different approaches and modifications enhancing their adsorption capacities. These methods not only contribute to improved water quality but also address the environmental concerns associated with dye pollution, highlighting their significance in sustainable water management practices [15,71,72,73]. One approach involves the use of thin-film composite nanofiltration membranes embedded with cellulose nanocrystals within the polyamide active layer [71]. These membranes exhibit excellent potential for desalination and dye removal, demonstrating high salt rejection rates and improved filtration flux. The modified surface charge and reduced pore size of these membranes enhance their effectiveness in removing both anionic and cationic dyes, providing a versatile solution for wastewater treatment [71].

Additionally, water-stable membranes composed of carboxymethyl cellulose (CMC) and cellulose nanofibrils (CNFs), cross-linked with citric acid, offer a promising alternative for dye removal [72]. These membranes maintain high dye removal efficiency over multiple reuse cycles, making them sustainable and cost-effective options for filtration technology. Phosphonated cellulose acetate (PCA) membranes have also shown promise in the adsorptive removal of methylene blue (MB) from water [15]. With a maximum adsorption capacity of 10 mg/g, these membranes demonstrate effective dye removal while being thermodynamically favorable and feasible for practical applications in water purification [15].

Furthermore, nanocomposite membranes composed of cellulose nanocrystals (CNCs) derived from microcrystalline cellulose are highly effective in removing cationic dyes from water [73]. The efficiency of dye removal depends on the concentration of CNCs used in membrane production, allowing for customization to meet specific filtration requirements. Another innovative approach involves modifying cellulose filter membranes with poly(diallyl dimethyl ammonium chloride) (PDADMAC) for the selective removal of anionic dyes from wastewater [74]. These membranes exhibit high selectivity in removing methyl orange (MO) and also show effective antimicrobial properties against *S. aureus* and *E. coli*, making them valuable for wastewater treatment applications [74].

The development and integration of advanced membrane materials offer significant promise for efficient dye removal from wastewater, contributing to improved water quality and environmental sustainability. These innovative approaches address the challenges associated with dye pollution, emphasizing their importance in modern water treatment technologies [75]. Modified cellulose nanocrystals (MCNC) have been integrated into polyethersulfone (PES) membranes to improve the water purification efficiency [76]. These membranes, with amine-functionalized cellulose nanocrystals, demonstrate excellent adsorption capabilities for both copper ions and direct red-16 dye from water. Including 1 wt% MCNC in PES membranes significantly increases the dye removal efficiency, reaching up to 99%, highlighting the effectiveness of MCNC in enhancing membrane performance [76]. Another notable approach involves the development of nanoporous membranes doped with silver nanoparticles (AgNPs) for the catalytic decolorization of dyes [77]. Using a bacterial cellulose (BC) hydrogel as a scaffold, the AgNP-doped BC membrane demonstrated exceptional efficiency in breaking down organic dyes like rhodamine 6G and methyl orange. Furthermore, its unique nanoporous structure ensures high decolorization efficiency, with minimal performance degradation over multiple cycles, demonstrating its potential for continuous dye treatment in water purification processes [77].

Additionally, a nanofiber composite nanofiltration membrane was created by grafting carboxyl multi-walled carbon nanotubes onto bacterial cellulose and coating the surface with a chitosan hydrogel. This membrane was designed for the highly efficient removal of dyes in wastewater treatment. The composite membrane showed improved performance at higher pressure and maintained a dye rejection rate above 90% for dyes heavier than 600 g/mol under 0.5 MPa. It also demonstrated effective anti-fouling properties against oil and proteins [78]. Another significant advancement in water filtration technology is the integration of ultrathin graphene oxide (GO)–cellulose nanofiber (CNF) composite membranes [79]. A membrane with a GO:CNF ratio of 1:100 exhibited remarkably high water flux. The anisotropic layers of the membrane efficiently rejected over 90% of charged dyes by combining electrostatic and hydrophobic interactions and size exclusion mechanisms. The GO layer on the CNF allows for the manufacture of cost-effective and scalable water purification membranes with excellent performance in terms of flux, stability, and dye rejection [79].

The use of cellulose acetate and its derivatives in various membrane configurations demonstrates their potential for the effective removal of dyes from wastewater. These innovative approaches address the challenges associated with dye pollution, contributing to improved water quality and environmental sustainability [80,81,82,83]. Cellulose acetate (CA) and activated carbon (AC) composite membranes have shown high adsorption efficiency for specific dyes [82]. Including 15% AC in these membranes resulted in significant adsorption capacities for acid blue, acid yellow, and acid dark dyes, making them promising options for targeted dye removal from solutions [82]. Moreover, CA-based nanocomposite films have been effective as adsorbents for the removal of methylene blue (MB) dye from wastewater streams [80]. By incorporating Cloisite 30B modified with polyacrylic acid (C30B-g-PAA) into CA films, the adsorption capacity for MB was enhanced, with a notable improvement observed at 50 wt% loading of C30B-g-PAA. This enhancement underscores the potential of CA-based nanocomposite films as efficient and reusable adsorbents for cationic dyes like MB [80]. Similarly, a study highlighted the highly efficient removal of methylene blue dye from an aqueous solution using cellulose acetate nanofibrous membranes modified by polydopamine [84]. Additionally, mixed-matrix membranes (MMM) composed of CA, graphene oxide (GO) nanosheets, and Fe_3_O_4_ magnetic nanoparticles offer efficient dye removal capabilities [81]. Through a phase inversion process, the nanocomposite membranes exhibited significant improvements in the rejection rates for anionic dyes such as acid blue 7, reactive red 120, and direct red 23, achieving up to 100% rejection at a pH of 9. This substantial enhancement over pure CA membranes highlights the effectiveness of the nanocomposite additive in enhancing the membrane functionality for water purification applications [81].

A study investigated the potential of two fully cellulose-based membranes as adsorbents for cationic dyes. The membranes were composed of cellulose nanofibrils (CNFs) and carboxymethylated cellulose (CMC), and they were compared with commercial bacterial cellulose (BC) membranes. The CNF/CMC-based membranes showed approximately 100% dye removal efficiency, while the BC and sodium periodate-treated form (BCox) membranes exhibited lower removal rates (60). Another study incorporated microfibrillated cellulose (MF–CNCs) into membranes, resulting in improved wet strength and enhanced dye removal efficiency [85].

**Figure 4:**
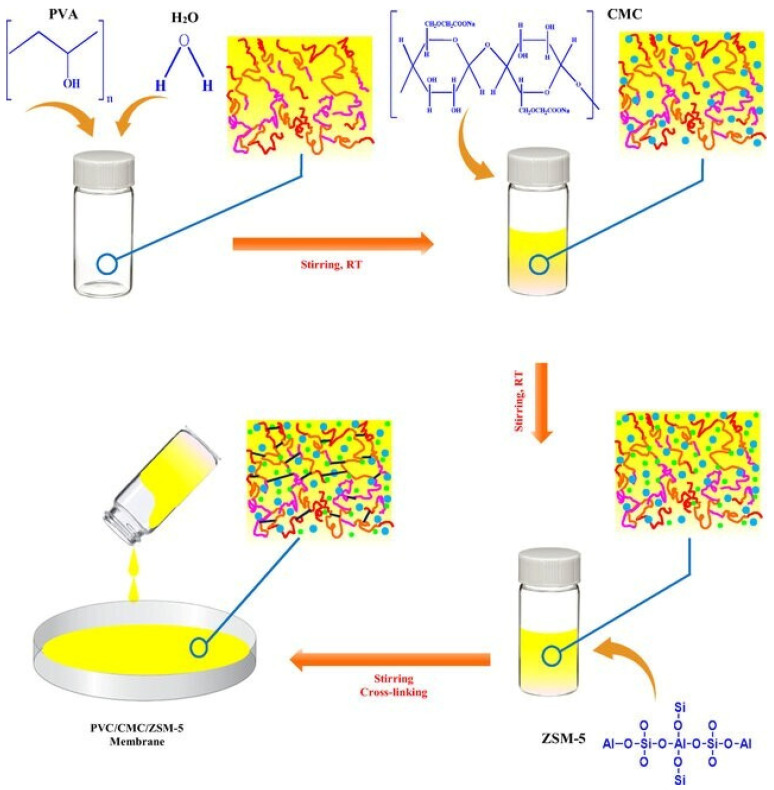
Cross-linking mechanism of PVA/CMC/ZSM-5 zeolite membranes for removal of methylene blue [86].

Additionally, a biodegradable membrane composed of polyvinol alcohol (PVA), carboxymethyl cellulose (CMC), and ZSM-5 zeolite was found to effectively remove methylene blue (MB). The study found that increased zeolite and dye concentrations improved the adsorption, while higher temperatures reduced it. A membrane containing 5 wt% zeolite achieved 97% removal efficiency and an adsorption capacity of 7.83 for a 10 ppm dye solution over 10 h at 30 °C (Figure 4) [86].

Chitosan/carboxymethyl cellulose (CTS/CMC) hollow capsules have demonstrated potential applications in dye wastewater treatment and controlled drug delivery systems. Capsules with molar ratios of 1/1 and 1/1.5 were prepared and effectively removed various dyes, including methylene blue, methyl orange, and acid blue 113. The porous structure of the CTS/CMC capsules showed selective permeability to methyl orange and allowed for the release of the absorbed dyes at varying rates [87]. Moreover, porous cellulose nanocrystals (CNCs) derived from Crotalaria juncea bast fibers have been found to effectively remove dyes. The adsorption of methylene blue by CNC is most efficient at a neutral pH, lower methylene blue concentration, and higher loading. Surface modification with 2,2,6,6-tetramethylpiperidinyloxy (TEMPO) was carried out on both pre-treated CJ and CNCs to enhance the adsorption efficiency. The modification decreased the methylene blue adsorption but increased the malachite green (MG) removal to 92%. The variation in the adsorption performance of CNCs and TEMPO CNCs is attributed to the varying charge density, surface area, and stereochemistry of both the adsorbate and the adsorbent, being the major factors in regulating adsorption in the case of unmodified CNCs, and the presence of radicals that are electronic in nature, dominating in the case of TEMPO CNCs [88].

## 5. Removal of Other Contaminants

In the future, the integration of advanced materials and innovative methods holds great potential in addressing water quality challenges and ensuring sustainable water management practices. Several studies have achieved significant progress in water treatment technology, offering promising solutions for the removal of pollutants and environmental remediation [89,90,91]. A comprehensive study was conducted that included various innovative approaches for water treatment and pollutant removal, showcasing significant advancements in the field. Firstly, a high-performance adsorptive electrospun nanofiber membrane composed of polyacrylonitrile nanofibers embedded with nanoclay showed exceptional efficacy in removing organic micropollutants from water. The addition of nanoclay led to a remarkable seven-fold increase in the adsorption capacity compared to standard polyacrylonitrile nanofibers. Optimal adsorption was achieved at pH 7 and 25 °C, with the Ads-ESNs maintaining excellent performance across multiple regeneration cycles, underscoring the effectiveness of integrating adsorption with membrane filtration [90].

Dispersing cellulose nanofibers (CNFs) in hydrophobic polymers like poly(lactic acid) (PLA) presents a significant challenge that hinders the broader application of cellulose nanocomposites. In another innovative approach, the solvent evaporation method, commonly employed in drug microencapsulation, was used to disperse poly(lactic acid) microparticles in water. This suspension of microparticles was then easily blended with CNFs produced through high-pressure homogenization. The removal of water via membrane filtration led to the formation of CNF sheets embedded with the microparticles. Subsequently, compressing these stacked sheets resulted in nanocomposites exhibiting the effective dispersion of the CNFs. The findings indicated remarkable enhancements in the modulus and strength—up to 58% and 210%, respectively—showcasing the impressive load-bearing capacity of the CNF network within the composites [91].

Furthermore, a cellulose acetate/zeolite fiber adsorbent showed remarkable efficiency in the removal of erythromycin from wastewater, with the maximum adsorption capacity recorded at 17.76 mg/g. The exothermic adsorption process, characterized by the change in enthalpy *(ΔH)* values, indicated high stability and potential for repeated use without environmental contamination after three cycles [89]. Moreover, cellulose acetate mixed-matrix nanofiltration membranes, embedded with functionalized silica nanoparticles, exhibited enhanced pharmaceutical rejection in removing the ceftriaxone sodium antibiotic from water. The membranes rejected approximately 90% of ceftriaxone sodium and 96% of CA/modified silica at pH 8, owing to size exclusion and charge repulsion mechanisms. Modification with (3-aminopropyl)triethoxysilane (APTES) and 2-acrylamido-2-methyl-1-propanesulfonic acid (AMPS) improved the membrane’s ability to remove anionic substances, promising significant advancements in water purification [92]

Furthermore, polymer inclusion membranes (PIMs) composed of the β-cyclodextrin polymer, polyvinyl chloride (PVC), and dibutyl phthalate (DBP) have shown potential for drug extraction from contaminated water. Interactions between the DBP and polymer chains increased the spacing within the PVC/poly(β-cyclodextrin) framework, enhancing molecule capture within cyclodextrins. Increased β-cyclodextrin polymer content and agitation improved the drug extraction efficiency, particularly in acidic conditions, highlighting their utility in water treatment [93]. Additionally, the effectiveness of recycled polyvinyl chloride (PVC) microplastics in adsorbing common antibiotics such as ciprofloxacin and clindamycin was investigated. With maximum adsorption capacities of 45.9 mg/g for ciprofloxacin and 22.0 mg/g for clindamycin, recycled PVC microplastics demonstrated notable potential in treating contaminated water, offering a sustainable solution for antibiotic removal [94].

## 6. Challenges and Limitations of Cellulose-Based Membranes

Cellulose-based membranes for wastewater treatment face several challenges and limitations, significantly affecting their efficiency and applicability. Cellulose membranes are particularly prone to fouling due to their surface hydrophobicity, which reduces their separation efficiency and flux. Fouling can lead to a decline in the membranes’ overall performance, necessitating more frequent cleaning and maintenance, which can be costly. One of the primary limitations is the tendency of cellulose materials to absorb water, which adversely affects their mechanical properties. This absorption can lead to a compromised structure under continuous operational conditions, potentially resulting in membrane failure [34]. Cellulose membranes typically exhibit poor chemical and thermal resistance, making them unsuitable for environments with harsh conditions. This limitation restricts their use in various industrial applications where aggressive chemicals and high temperatures are involved. Improving the selectivity of cellulose membranes for specific molecules often requires modifications that can be technically challenging and economically unviable. The balance between enhancing selectivity and maintaining the membranes’ other desirable properties, such as permeability, is complex and requires extensive research and development. The durability of cellulose membranes under continuous operation is another concern, as their performance can deteriorate over time due to wear and environmental factors. This impacts their long-term viability in wastewater treatment applications [95].

While cellulose materials offer many benefits, their inherent properties may not meet all application requirements. Thus, extensive modifications are often necessary to enhance their performance characteristics, which can complicate the production process and increase the costs [96]. The performance of cellulose-based membranes can be influenced by environmental factors such as the temperature, pH, and the presence of specific contaminants. Variability in these conditions can result in inconsistent treatment efficiencies, making standardization across applications difficult [97]. Cellulose-based membranes hold promise for wastewater treatment due to their environmentally friendly nature; however, overcoming these challenges is crucial for their effective implementation in various settings.

## 7. Conclusions and Future Prospects

This review discusses various innovative approaches and materials for the removal of contaminants from water, including antibiotics, dyes, and other pollutants. These approaches include techniques such as membrane filtration, adsorption, photocatalysis, and nanocomposite materials, demonstrating the diverse and versatile methods available for water treatment. The use of advanced materials like cellulose nanocrystals, metal–organic frameworks, and graphene-based composites in membrane technologies has significantly improved the adsorption capacities, selectivity, and efficiency for contaminant removal. Furthermore, the development of novel membrane configurations, such as cellulose acetate nanofiber membranes and hybrid nanocomposite membranes, shows promise in improving water purification processes. Many of the discussed approaches utilize biodegradable materials, green synthesis methods, and recyclable sorbents, reflecting a commitment to environmentally friendly solutions.

Addressing key research areas and challenges in water treatment can lead to the provision of clean and safe water for communities worldwide. Future advancements in water treatment technologies should focus on several key areas to address emerging challenges and improve overall efficiency. Combining different treatment processes, such as membrane filtration, adsorption, and photocatalysis, in integrated systems could enhance the overall efficiency and address a wider range of contaminants. Developing multifunctional materials with capabilities for adsorption, photocatalysis, and antimicrobial activity could lead to more versatile and efficient water treatment solutions. It is crucial to assess the long-term environmental impacts of water treatment technologies, including the fate of the removed contaminants and potential secondary pollution sources. While many innovative materials show promising results at the lab scale, their scalability and cost-effectiveness for large-scale water treatment applications need further investigation and optimization.

## Data Availability

Not applicable.
